# Combined Medial Patellofemoral Ligament Reconstruction and Tibial Tubercle Osteotomy Has a Lower Risk of Recurrent Instability Requiring Revision Stabilization at 2 Years Than Either Procedure Alone

**DOI:** 10.1016/j.asmr.2024.100994

**Published:** 2024-09-06

**Authors:** Alexander R. Markes, Ramesh B. Ghanta, Alan L. Zhang, C.Benjamin Ma, Brian T. Feeley, Drew A. Lansdown

**Affiliations:** Department of Orthopaedic Surgery, University of California-San Francisco, San Francisco, California, U.S.A.

## Abstract

**Purpose:**

To use a large nationwide administrative database to directly compare usage, complications, and need for revision stabilization surgery after medial patellofemoral ligament reconstruction (MPLFR), tibial tubercle osteotomy (TTO), and combined MPFLR and TTO (MPFLRTTO).

**Methods:**

The PearlDiver Mariner database was queried for all reported cases of MPLFR, TTO, and combined MPFLRTTO performed between 2010 and 2020 using Current Procedural Terminology codes. Subsets from those cohorts with laterality-specific *International Classification of Diseases*, *Tenth Revision*, codes for patellar instability were used to evaluate 2-year incidence of infection, stiffness, fracture, and revision stabilization with MPFLR and/or TTO. Multiple linear regression and χ^2^ analysis were used to analyze incidence trends and to compare complication rates.

**Results:**

A total of 70,070 patients were identified. MPFLR was found to be the most common procedure (73.1%), followed by TTO (19.2%) and then MPFLRTTO (7.6%). MPLFR was observed to have the lowest overall complication rate (5.4%), whereas both TTO (7.5%) and MPFLRTTO (7.1%) had greater complication rates (*P* < .001). MPFLR had the greatest rate of revision stabilization surgery at 3.7% compared with TTO at 2.7% and MPFLRTTO, which carried the lowest risk for revision at 2.4% (*P* < .001).

**Conclusions:**

Isolated MPFLR is the most common modality used for patellar instability, with increasing prevalence and the lowest 2-year complication rate. Isolated TTO was unchanged in its use and had the greatest overall complication rate. Combined MPFLRTTO increased the overall complication rate but had a lower 2-year rate of recurrent instability requiring revision than MPFLR alone.

**Level of Evidence:**

Level III, retrospective cohort study.

Patellofemoral instability is a common condition that predominantly affects young and active patients, leading to considerable limitations in physical activity, an increased risk of recurrent instability/dislocation, and early-onset patellofemoral arthritis.^1^, ^2^, ^3^, ^4^, ^5^ The etiology of patellar instability can be multifactorial, ranging from an isolated tear of the medial patellofemoral (MPFL) ligament or as a result of several additional anatomic variables, including trochlear dysplasia, elevated tibial tubercle-trochlear groove distance (TT-TG), patella alta, or excessive femoral anteversion.^6^^,^^7^ Although first-time patellar dislocations are generally treated conservatively, there is a high risk of recurrent dislocation and instability, with operative intervention often the best management option for cases of chronic instability.^8^^,^^9^

Given this complex and multifactorial etiology of patellar instability, several procedures are performed to restore stability to the patellofemoral joint, including medial patellofemoral reconstruction (MPFLR), distal realignment of the extensor mechanism with a tibial tubercle osteotomy (TTO), or a combination of both procedures (MPFLRTTO). Procedures to alter the shape of the trochlear, such as trochleoplasty, also can be performed. As our understanding of the role of soft-tissue tensioning and bony anatomy–related patellofemoral instability has improved, MPFLR with or without TTO has become a reliable surgical option to manage this pathology.

Several clinical studies have demonstrated the ability of MPFLR, TTO, or combined procedures to reduce rates of patellar instability and improve knee function.^10^, ^11^, ^12^, ^13^, ^14^, ^15^ Increasing interest in combined ligamentous and bony procedures has been spurred by concerns that bony anatomic considerations affect the success of isolated ligamentous procedure. In contrast, there are numerous studies demonstrating that isolated MPFL can be adequate in treating patellar instability regardless of bony anatomy. In their meta-analysis, Guevel et al.^16^ demonstrated no significant differences in redislocation rates between isolated MPFLR and combined MPFL and TTO in patients with TT-TG >20. Similarly, Vivekanantha et al.^17^ demonstrated similar redislocation rates in patients with an elevated TT-TG after isolated MPLFR compared with concomitant MPFLR and TTO. Erickson et al.^18^ demonstrated no self-reported patellofemoral instability with an 88% 2-year return to sport rate in 99 consecutive patients with patellofemoral instability treated with isolated MPFLR regardless of TT-TG distance, Caton-Deschamps index, and trochlear dysplasia. In addition, a survey of the International Patellofemoral Study Group in 2018 did not come to a consensus regarding indications for bony procedures to augment MPFLR.^19^ Despite isolated MPFLR being an option for treatment of patellar instability even in the setting of altered bony anatomy, an update is needed regarding management and risk for revision surgery to assist in preoperative decision making.

The goal of this study is to use a large nationwide administrative database to directly compare use, complications, and need for revision stabilization surgery after isolated MPFLR, isolated TTO, or combined MPFLR and TTO. We hypothesized that despite isolated MPFLR being the most common procedure, we would expect increasing use of combined MPFLR and TTO for patellar instability and that combined MPFLR and TTO would have a greater overall complication rate although the lowest rate of recurrent instability compared with isolated MPFLR or TTO.

## Methods

This analysis was performed using the PearlDiver Mariner All-Payer Claims Database (Colorado Springs, CO), a retrospective nationwide insurance billing database that provides deidentified and patient-specific claims.^20^ The subset used for this analysis was a random subset including 157 million patients. The PearlDiver database includes claims from patients of all age groups across the United States that are enrolled with various private payer commercial insurances or Medicare advantage plans from 2010 through 2020. Although deidentified and compliant with the Health Insurance Portability and Accountability Act, this dataset is also capable of longitudinal research on the basis of unique patient identifier codes. This database allows for searching of patients with any *International Classification of Diseases*, *Tenth Revision* (ICD-10), or Current Procedural Terminology (CPT) code.^21^, ^22^, ^23^ It has been used in previous population-scale analyses both in patellar instability analysis and other orthopaedic surgery procedures.^21^^,^^24^ This work is institutional review board exempt, given this was a retrospective review with no identifying patient data accessed.

### Inclusion Criteria

All reported cases of MPFLR, TTO, or combined MPFLRTTO performed between 2010 and 2020 were queried from the database using CPT codes (Table 1). The CPT codes 27420 (Reconstruction of dislocating patella), 27422 (Reconstruction of dislocating patella with extensor realignment and/or muscle advancement or release), and 27427 (Ligamentous reconstruction (augmentation), knee; extra-articular) were used to query the database for MPFLR. The code 27418 (Anterior tibial tubercleplasty) was used to query the database for TTO. The MPFLR and TTO cohort was defined as patients who had CPT codes for any of the 3 MPFLR procedures and the CPT code for TTO on the same day. Patients who underwent previous MPFLR were excluded from analysis. Demographics recorded to describe our cohort included year of surgery, sex, Charlson Comorbidity Index, obesity, patient-reported tobacco use, diabetes, and patient age at the time of primary patellar stabilization procedure.

**Table 1:** Current Procedural Terminology (CPT) Codes of Primary Patellar Stabilization Surgeries, Reoperations, and Complications

Medial patellofemoral ligament reconstruction	27420 – Reconstruction of dislocating patella (e.g., Hauser-type procedure)
	27422 – Reconstruction of dislocating patella with extensor realignment and/or muscle advancement or release
	27427 – Ligamentous reconstruction (augmentation), knee; extra-articular
Tibial tubercle osteotomy	27418 – Anterior tibial tubercleplasty
Infection	27301 – Incision and drainage, deep abscess, bursa, or hematoma, thigh or knee region
	27303 – Incision, deep, with opening of bone cortex, femur or knee (e.g., osteomyelitis or bone abscess)
	27310 – Arthrotomy, knee, with exploration, drainage, or removal of foreign body (e.g., infection)
	29871 – Arthroscopy, knee, surgical; for infection, lavage, and drainage
Stiffness	27570 – Manipulation of knee joint under general anesthesia
	29884 – Arthroscopy, knee, surgical; with lysis of adhesions, with or without manipulation
Fracture	27520 – Closed treatment of patellar fracture, without manipulation
	27524 – Open treatment of patellar fracture, with internal fixation and/or partial or complete patellectomy and soft tissue repair
	27792 – Fracture and/or dislocation procedures on the leg (tibia and fibula) and ankle joint
	27530 – Closed treatment of tibial fracture proximal (plateau); without manipulation
	27540 – Open treatment of intercondylar spine(s) and/or tuberosity fracture(s) of the knee with or without internal or external fixation
Other	29874 – Arthroscopy, knee, surgical; for removal of loose body or foreign body
	29877 – Arthroscopy, knee, surgical; debridement/shaving of articular cartilage (chondroplasty)
	29879 – Arthroscopy knee, surgical; abrasion arthroplasty (includes chondroplasty where necessary) or multiple drilling or microfracture

### Revision Analysis

For the 2-year revision surgery analysis, only patients with laterality-specific ICD-10 codes (Appendix Table 1, available at www.arthroscopyjournal.org) for patellar instability linked to the same day as the CPT procedure code for either MPFLR, TTO, or combined MPFLRTTO were analyzed. This subset of patients then was tracked for 2-year incidence of repeat surgery for infection, stiffness, fracture, and revision stabilization with isolated MPFLR or revision stabilization with isolated TTO using CPT codes listed in Table 1 linked to a laterality specific ICD-10 code for infection, fracture, stiffness, or patellar instability (Appendix Table 1). ICD-10 coding allows for laterality-specific tracking to ensure that revision procedures were performed on the ipsilateral side as the index procedure. PearlDiver allows for “active” tracking of patients, which confirms they maintained insurance enrollment and follow-up with a provider during a specified time period. This function was used as a proxy for ensuring patients were not lost to follow-up during the 2-year postoperative window of analysis.

### Statistical Analysis

All graphing and statistical analyses were performed using Microsoft Excel, version 16.46 (Microsoft Excel XLSTAT, New York, NY). The change in annual patellar stabilization procedures performed from 2010 to 2020 was analyzed using multiple linear regression. Overall fit of the model was evaluated through F-statistic, degrees of freedom, and its significance (*P* < .05). χ^2^ analysis was used to compare incidence of 2-year complications among primary patellar stabilization procedures. Significance was defined as *P* < .05.

## Results

**Fig 1:**
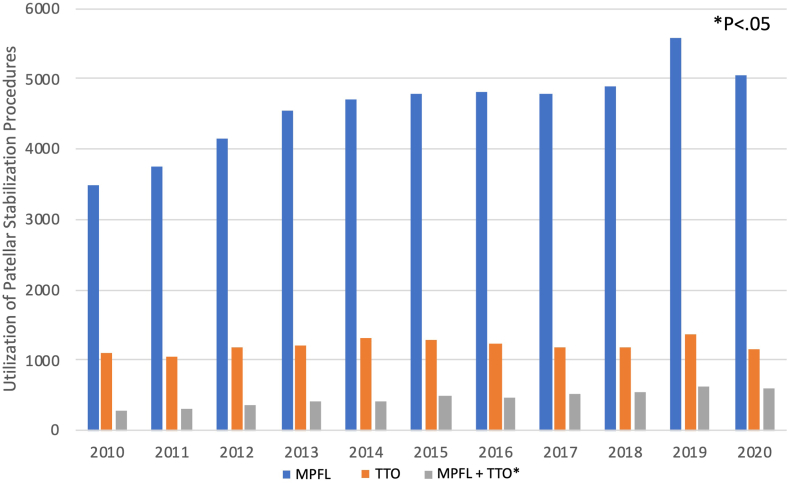
Annual incidence of patellar stabilization procedures. *P* < .05 is in reference to *P* value generated from the multiple linear regression model analyzing change in annual patellar stabilization procedure performed. (MPFL, medial patellofemoral ligament reconstruction; MPFL + TTO, concomitant medial patellofemoral ligament reconstruction and tibial tubercle osteotomy; TTO, tibial tubercle osteotomy.)

A total of 70,070 patients undergoing patellar stabilization procedures from 2010 to 2020 and who met the aforementioned inclusion criteria were identified. Of these patients, 51,238 (73.1%) underwent isolated MPFLR, 13,483 underwent isolated TTO (19.2%), and 5,349 (7.6%) underwent combined MPFL and TTO. There was an increased incidence of all 3 procedures in younger age demographics, with the greatest incidence in those aged 10 to 19 years. All 3 procedures were more predominant in female patients, with differences in obesity, diabetes, and tobacco use noted in Table 2. The multiple linear regression model evaluating change in annual patellar stabilization procedures was significant (F[3,7] = 47.6, adjusted R^2^ = 0.97, *P* < .001) with a significant increase in MPFLRTTO procedures per year (β = 0.51, *P* = .023). Although MPFL procedures performed increased from 3,485 to 5,047 (β = –0.02, *P* = .396) and TTO procedures increased from 1,097 to 1169 (β = –0.09, *P* = .231) over the study period, these changes were not significant in the multiple linear regression model (Fig 1).

**Table 2:** Patient Demographics for Stabilization Procedures

	MPFL (n = 51,238)	TTO (n = 13,483)	MPFL + TTO (n = 5,349)	*P*
Age group, yr				–
10-19	41%	35%	47%	
20-29	22%	26%	28%	
30-39	15%	22%	17%	
40-49	9%	13%	7%	
50-59	6%	4%	2%	
60-69	5%	1%	1%	
70+	4%	1%	0.2%	
CCI (SD)	0.69 (1.3)	0.62 (1.1)	0.53 (0.9)	–
Female	64%	77%	75%	<.01∗
Obesity	34%	36%	33%	<.01∗
Diabetes	15%	14%	9%	<.01∗
Tobacco use	26%	27%	23%	<.01∗

CCI, Charlson Comorbidity Index; MPFL, medial patellofemoral ligament reconstruction; MPFL + TTO, concomitant medial patellofemoral ligament reconstruction and tibial tubercle osteotomy; TTO, tibial tubercle osteotomy.

∗ Denotes *P* < .05 for χ^2^ analysis between all patellar stabilization procedures and demographic variable of respective row of table.

We identified a subset of 19,627 patients (Table 3) who underwent patellar stabilization surgery with a same-day laterality specific ICD-10 code for patellar instability linked to the CPT code for the patellar stabilization for our revision analysis. Of those patients, the index patellar stabilization procedure was MPFLR for 14,436, TTO for 3,425, and combined MPFLRTTO for 1,766. MPFLR was observed to have the lowest overall complication rate (5.4%), whereas both TTO (7.5%) and MPFLRTTO (7.1%) had greater complication rates (*P* < .001). With respect to revision surgery for recurrent instability, MPFLR was noted to have the greatest rate at 3.7% compared with TTO which was 2.7% and combined MPFLRTTO, which carried the lowest risk for revision at 2.4% (*P* < .001). Overall rates of fracture and infection rate were low (<1%), with MPFLR having the greatest rate of infection but the lowest risk of fracture. There was no significant difference in rates of revision surgery for stiffness between groups.

**Table 3:** Two-Year Complications and Revisions Performed Per Index Operation

Indication for Reoperation	MPFL (n=14,436)	TTO (n=3,425)	MPFL + TTO (n=1,766)	*P*
All complications	5.4%	7.5%	7.1%	<.01∗
Infection	0.9%	0.4%	0.6%	.01∗
Stiffness	2.8%	3.2%	3.6%	.13
Fracture	0.3%	0.7%	0.7%	.01∗
Instability requiring isolated MFPL	3.4%	1.8%	2.2%	<.01∗
Instability requiring isolated TTO	0.9%	1.4%	1.0%	.03∗
Instability requiring MPFL or TTO	3.7%	2.7%	2.4%	<.01∗

MPFL, medial patellofemoral ligament reconstruction; MPFL + TTO, concomitant medial patellofemoral ligament reconstruction and tibial tubercle osteotomy; TTO, tibial tubercle osteotomy.

∗ Denotes *P* < .05 for χ^2^ analysis between all patellar stabilization procedures and complication or revision of respective row of table.

## Discussion

MPFLR was observed to be the most common surgical option for treating patellar instability in this population-wide database study of patients. The lowest rates of recurrent instability were observed in combined MPFLRTTO, whereas this cohort also had the greatest rates of postoperative complications. The use of all-patellar stabilization procedures has increased over the past 10 years with the increase in combined MPFLR and TTO increasing most linearly per year.

The treatment of patellar instability has evolved in recent years, as there is continued recognition of the appropriate procedures needed to address this complex pathology. Previously, a study on this topic included 6,190 patients between 2007 and 2014 and noted a significant increase in annual number of patellar instability procedures performed using similar CPT codes to our study. However, they did not break up annual use of their concomitant procedures.^24^ In our study, we observed an increased incidence of both isolated MPFLR and combined MPFLRTTO. This observation may reflect improved surgeon familiarity with the techniques of MPFL reconstruction and increased understanding of reliable success of reconstruction with or without TTO to be further discussed in this work.^24^

With respect to complications, our data showed a low overall complication rate for each of the 3 procedures, with isolated soft-tissue reconstruction associated with a lower overall complication rate than isolated or concomitant bony procedures in this patient cohort. Specifically, for MPFLR, complication rates within the literature vary widely. Howells et al.^25^ looked at 219 MPFLR procedures and demonstrated a complication rate of 3.3%, whereas a larger systematic review of 629 procedures conducted by Shah et al.^12^ in 2013 reported a complication rate of 26.1%. Jackson et al.^26^ reported a wide range of complications of MPFLR in their systematic analysis with rates of overall complications ranging from 0 to 32.3%. When examining isolated or concomitant TTO complication rates, our data also appear to be similar to those reported in recent studies. In a systematic review of 787 isolated TTO procedures conducted by Payne et al.,^11^ the authors found an overall complication rate of 4.6%. In a retrospective review of 59 concomitant MPFLR/TTO procedures, Markus et al.^27^ found a complication rate of 10.1%. The discrepancy in complication rates within our large national database and across the literature can likely be attributed to significant variations in inclusion criteria for complications. For example, in our study, with an MPLFR complication rate of 5.4%, we chose to focus only on complications requiring repeat operation compared with the meta-analysis by Shah et al.^12^ (MPFLR complication rate of 26.1%) in which complications included a broader range of issues such as “patellar apprehension,” “persistent pain,” and “mild patellar hypermobility.” Subjective symptomatic complaints like these cannot be discerned with the current database design, although the 5.4% observed complication rate can be useful for surgeons when counseling patients regarding more significant complications that may require additional treatment.

Using a national administrative database, we were able to analyze the incidence of revision surgery after each of the 3 primary procedures in a large patient cohort. The potential risk of revision surgery should be balanced against the risk of recurrent instability, and the results of this study provide important information that surgeons may use when making treatment recommendations. With our ability to specify laterality of procedure, which allows for reliable connection between primary and subsequent procedures, our results show that patients undergoing primary MPFLR experienced a 3.7% rate of revision with either MPFL or TTO whereas those undergoing TTO or concomitant procedures had revision rates of 2.7% and 2.4%, respectively. These rates are fairly consistent with the literature, which show rates of instability of 0.9% to 10% after MPFLR,^12^^,^^28^^,^^29^ 0 to 7.4% after TTO,^30^ and 0 to 5.6% after combined MPFLTTO.^10^^,^^27^^,^^29^^,^^31^, ^32^, ^33^ Previous work by Arshi et al.^24^ has demonstrated rates of infection, knee stiffness, and infection after primary patellar stabilization surgery, but given limitations in their database, were unable to identify the rate of revision for any specific procedure. The data from the current study may provide better insight to specific risks of further surgical intervention.

Instability requiring revision surgery is a complication with a likely multifactorial etiology, comprising both anatomic factors such as patella alta or trochlear dysplasia as well as surgical factors such as graft healing and/or tunnel placement.^6^^,^^7^^,^^12^ Previous studies have linked a tibial tubercle to trochlear groove (TT-TG) distance of greater than 20 mm with the need for TTO to properly address instability.^6^^,^^15^ Franciozi et al.^15^ in a prospective review of isolated MPFLR versus combined MPFLRTTO in patients with TT-TG 17-20 found improved functional outcome scores and clinically tested patellar kinematics at a mean 40 months postoperatively. However, this observation is tempered by conflicting studies that even in the setting bony abnormalities such as an elevated TT-TG distance isolated MPFLR can lead to equivalent outcomes compared with combined procedures.^18^ A systematic review by Vivekanantha et al.^17^ of more than 1,400 patients with patella instability and TT-TG indices of greater than 15 mm showed isolated MPLFR had similar redislocation rates (3.1% vs 3.2%), similar Kujala anterior knee pain scores (85.0 vs 83.7), but lower return to sport (82% vs 92%) compared with combined MPFLR and TTO. Our analysis showed a significant difference between revision instability for MPFL, TTO, and MPFLRTTO, although the rate of revision for instability for MPFL (3.7%) and MPFLRTTO (2.4%) is similar to the rate of redislocation reported by Vivekanantha et al.^17^ These differences in what is viewed as significant despite having similar rates are likely the result of the larger sample size of our study, which can be more sensitive for small differences in outcomes, although whether these differences are clinically significant to our patients is still unknown.

Despite MPFLRTTO having the lowest revision rate, which patients would benefit most from isolated MPFLR and which may benefit more from the addition of a concomitant procedure is still undefined. The use of radiographic measurements such as the TT-TG or the tibial tubercle-posterior cruciate ligament have been used to assess the amount of lateralization of the tibial tubercle though cutoffs used by surgeons vary, and, as stated previously, isolated MPFLR still is a viable option for good outcomes.^17^, ^18^, ^19^ Other factors that have been theorized to place patients at risk for recurrent instability include patellar height, trochlear dysplasia, and patellar type. Kita et al.^34^ analyzed these and other factors in their 42 patients who underwent isolated MPFLR for patellar instability regardless of bony or alignment abnormalities. At 2-year follow-up, they noted trochlear dysplasia was the only variable independently associated with postoperative patellofemoral instability and that TT-TG distance exerted a significant effect on the outcomes of MPFL reconstruction, particularly in patients with type D trochlea. Additional stabilization procedures such as a TTO or trochleoplasty may play a role in these patients. However, it is important to note the study had 11 of 42 patients with Dejour D trochlear dysplasia, although only 2 of 42 patients experienced redislocation meaning even a subset of their patients with Dejour D trochlea had an acceptable outcome with isolated MPFLR.^34^

Our data illustrated a low infection rate for all procedures, with incidence below 1% for all 3 subsets. Several studies analyzing both short-term 30 day infection rates as well as longer follow-up studies for these cohorts have consistently found the incidence of infection to range from 0% to 5%, which is in line with our data.^14^^,^^29^^,^^32^^,^^33^^,^^35^ With respect to fracture, the risk for all three procedures is less than 1%, which is similar to reports seen in the literature with Vivekantha et al.^17^ reporting a prevalence 0.6% after isolated MPFLR and a 0.4% after combine MPFLR and TTO. Jackson et al.^26^ reported an incidence of patellar fracture ranging from 0 to 8.3%, primarily in patients treated with full-length transverse tunnel or 2-tunnel techniques. Given the limitations of our database, we are unable to assess the exact surgical technique for patellar fixation of the graft. With respect to stiffness, patients undergoing isolated soft-tissue procedure appeared to have lower rates of stiffness than those undergoing bony procedures. Jackson et al.^26^ reported in their meta-analysis of studies looking at complications after MPLFR that 20 of the 28 studies had a 0% rate of postoperative stiffness requiring manipulation after MPFLR.^26^ It is likely that delayed return to weight-bearing and full-knee range of motion required after more extensive combined ligamentous and bony stabilizations procedures would contribute to increased risk of requiring secondary procedures to address residual stiffness.

### Limitations

This study has limitations. There is limited granularity within a publicly available database and as such we were unable to evaluate patient-level factors such as trochlear dysplasia for recurrent patellar instability which may point toward different indications for performing certain stabilization procedures. We did not extract data of all possible concomitant procedures at time of initial patellar stabilization surgery as such there could be unknown variables leading to increased risks of some of the complications evaluated. The selection of a TTO may be primarily done based on the presence of a lateralized tubercle with an elevated TT-TG. We are unable to control or account for the TT-TG, amongst other anatomic factors, which for some surgeons certainly play a role in determining a specific surgical plan. These differences may contribute to differences in revision rates, although as discussed previously, there is evidence that isolated soft tissue procedures even with bony abnormalities have been shown to be a viable option.^17^ In addition, although our selection of CPT codes was intended to be as comprehensive for complications and reoperations as possible, there is a possibility that procedures were billed under separate codes and thus not captured in our dataset. Another limitation inherent to the use of a large administrative database is the inability to confirm procedure aside from CPT procedure codes thus relying on accurate coding of procedures performed at the physician level. With respect to complications, our study only analyzed those which required subsequent surgery, thus not accounting for all complications nor functional clinical outcomes of these procedures.

## Conclusions

Isolated MPFLR is the most common modality used for patellar instability, with increasing prevalence and the lowest 2-year complication rate. Isolated tibial tubercle osteotomy was unchanged in its utilization and had the highest overall complication rate. Combined MPFLR and TTO increased the overall complication rate but had a lower 2-year rate of recurrent instability requiring revision than MPFLR alone.

## Disclosures

The authors declare the following financial interests/personal relationships which may be considered as potential competing interests: D.A.L. reports consulting or advisory with Vericel and AlloSource and educational support from Arthrex. B.T.F. reports Journal Editor, *Journal of Shoulder and Elbow Surgery* and *Current Review in Musculoskeletal Medicine*. C.B.M. reports consulting or advisory with Stryker and CONMED and a research grant from Aesculap. A.L.Z. reports consulting or advisory with Stryker, DePuy Synthes Mitek Sports Medicine, and CONMED. All other authors (A.R.M., R.G.) declare that they have no known competing financial interests or personal relationships that could have appeared to influence the work reported in this paper.

## References

[1] Kamalapathy P., K Rush J., Montgomery S.R., Diduch D.R., Werner B.C. (2022). A national perspective of patellar instability in children and adolescents in the United States: MPFL reconstruction is three times higher than the incidence of isolated lateral release. Arthroscopy.

[2] Hawkins R.J., Bell R.H., Anisette G. (1986). Acute patellar dislocations. Am J Sports Med.

[3] Clark D., Metcalfe A., Wogan C., Mandalia V., Eldridge J. (2017). Adolescent patellar instability: Current concepts review. Bone Jt J.

[4] Watson R., Sullivan B., Stone A.V. (2022). Lateral patellar dislocation: A critical review and update of evidence-based rehabilitation practice guidelines and expected outcomes. JBJS Rev.

[5] Koh J.L., Stewart C. (2015). Patellar instability. Orthop Clin North Am.

[6] Dejour H., Walch G., Nove-Josserand L., Guier Ch (1994). Factors of patellar instability: An anatomic radiographic study. Knee Surg Sports Traumatol Arthrosc.

[7] Dietrich T., Fucentese S., Pfirrmann C. (2016). Imaging of individual anatomical risk factors for patellar instability. Semin Musculoskelet Radiol.

[8] Rhee S.J., Pavlou G., Oakley J., Barlow D., Haddad F. (2012). Modern management of patellar instability. Int Orthop.

[9] Laidlaw M.S., Diduch D.R. (2017). Current concepts in the management of patellar instability. Indian J Orthop.

[10] Mulliez A., Lambrecht D., Verbruggen D., Van Der Straeten C., Verdonk P., Victor J. (2017). Clinical outcome in MPFL reconstruction with and without tuberositas transposition. Knee Surg Sports Traumatol Arthrosc.

[11] Payne J., Rimmke N., Schmitt L.C., Flanigan D.C., Magnussen R.A. (2015). The incidence of complications of tibial tubercle osteotomy: A systematic review. Arthroscopy.

[12] Shah J.N., Howard J.S., Flanigan D.C., Brophy R.H., Carey J.L., Lattermann C. (2012). A systematic review of complications and failures associated with medial patellofemoral ligament reconstruction for recurrent patellar dislocation. Am J Sports Med.

[13] Longo U.G., Berton A., Salvatore G. (2016). Medial patellofemoral ligament reconstruction combined with bony procedures for patellar instability: Current indications, outcomes, and complications. Arthroscopy.

[14] Agarwalla A., Gowd A.K., Liu J.N. (2019). Concomitant medial patellofemoral ligament reconstruction and tibial tubercle osteotomy do not increase the incidence of 30-day complications: An analysis of the NSQIP database. Orthop J Sports Med.

[15] Franciozi C.E., Ambra L.F., Albertoni L.J.B. (2019). Anteromedial tibial tubercle osteotomy improves results of medial patellofemoral ligament reconstruction for recurrent patellar instability in patients with tibial tuberosity–trochlear groove distance of 17 to 20 mm. Arthroscopy.

[16] Guevel B., Njai A., Raboff A., Hillman A., Barton M., Kocher M.S. (2023). Does tibial tuberosity osteotomy improve outcomes when combined with medial patellofemoral ligament reconstruction in the presence of increased tibial tuberosity–trochlear groove distance? A systematic review and meta-analysis. Orthop J Sports Med.

[17] Vivekanantha P., Kahlon H., Cohen D., de S.A.D. (2023). Isolated medial patellofemoral ligament reconstruction results in similar postoperative outcomes as medial patellofemoral ligament reconstruction and tibial-tubercle osteotomy: A systematic review and meta-analysis. Knee Surg Sports Traumatol Arthrosc.

[18] Erickson B.J., Nguyen J., Gasik K., Gruber S., Brady J., Shubin Stein B.E. (2019). Isolated medial patellofemoral ligament reconstruction for patellar instability regardless of tibial tubercle–trochlear groove distance and patellar height: Outcomes at 1 and 2 years. Am J Sports Med.

[19] Liu J.N., Steinhaus M.E., Kalbian I.L. (2018). Patellar instability management: A survey of the international patellofemoral study group. Am J Sports Med.

[20] Bolognesi M.P., Habermann E.B. (2022). Commercial claims data sources: PearlDiver and individual payer databases. J Bone Jt Surg.

[21] Markes A.R., Cevallos N., Lansdown D.A., Ma C.B., Feeley B.T., Zhang A.L. (2022). Risk for recurrent instability and reoperation following arthroscopic and open shoulder stabilization in a large cross-sectional population. JSES Int.

[22] Zhang A.L., Montgomery S.R., Ngo S.S., Hame S.L., Wang J.C., Gamradt S.C. (2013). Analysis of rotator cuff repair trends in a large private insurance population. Arthroscopy.

[23] Zhang A.L., Montgomery S.R., Ngo S.S., Hame S.L., Wang J.C., Gamradt S.C. (2014). Arthroscopic versus open shoulder stabilization: Current practice patterns in the United States. Arthroscopy.

[24] Arshi A., Cohen J.R., Wang J.C., Hame S.L., McAllister D.R., Jones K.J. (2016). Operative management of patellar instability in the United States: An evaluation of national practice patterns, surgical trends, and complications. Orthop J Sports Med.

[25] Howells N.R., Barnett A.J., Ahearn N., Ansari A., Eldridge J.D. (2012). Medial patellofemoral ligament reconstruction. J Bone Jt Surg.

[26] Jackson G.R., Tuthill T., Gopinatth V. (2023). Complication rates after medial patellofemoral ligament reconstruction range from 0% to 32% with 0% to 11% recurrent instability: A systematic review. Arthroscopy.

[27] Markus D.H., Hurley E.T., Gipsman A. (2022). Adding a tibial tubercle osteotomy with anteromedialisation to medial patellofemoral ligament reconstruction does not impact patient-reported outcomes in the treatment of patellar instability. J ISAKOS.

[28] Heyworth B.E., Zheng E.T., Hussain Z.B. (2021). Isolated MPFL reconstruction vs. Tibial tubercle osteotomy and medial retinacular plication for recurrent patellar instability: A matched, cohort analysis of clinical outcomes comparing two techniques. Orthop J Sports Med.

[29] Burnham J.M., Howard J.S., Hayes C.B., Lattermann C. (2016). Medial patellofemoral ligament reconstruction with concomitant tibial tubercle transfer: A systematic review of outcomes and complications. Arthroscopy.

[30] Palmer S.H., Servant C.T.J., Maguire J., Machan S., Parish E.N., Cross M.J. (2004). Surgical reconstruction of severe patellofemoral maltracking. Clin Orthop.

[31] Su P., Liu X., Jian N., Li J., Fu W. (2021). Clinical outcomes and predictive factors for failure with MPFL reconstruction combined with tibial tubercle osteotomy and lateral retinacular release for recurrent patellar instability. BMC Musculoskelet Disord.

[32] Watanabe T., Muneta T., Ikeda H., Tateishi T., Sekiya I. (2008). Visual analog scale assessment after medial patellofemoral ligament reconstruction: With or without tibial tubercle transfer. J Orthop Sci.

[33] Cossey A.J., Paterson R. (2005). A new technique for reconstructing the medial patellofemoral ligament. Knee.

[34] Kita K., Tanaka Y., Toritsuka Y. (2015). Factors affecting the outcomes of double-bundle medial patellofemoral ligament reconstruction for recurrent patellar dislocations evaluated by multivariate analysis. Am J Sports Med.

[35] Schöttle P., Fucentese S., Romero J. (2005). Clinical and radiological outcome of medial patellofemoral ligament reconstruction with a semitendinosus autograft for patella instability. Knee Surg Sports Traumatol Arthrosc.

