# Supplementary material for: Combined Medial Patellofemoral Ligament Reconstruction and Tibial Tubercle Osteotomy Has a Lower Risk of Recurrent Instability Requiring Revision Stabilization at 2 Years Than Either Procedure Alone
Source: Arthrosc Sports Med Rehabil. 2024 Sep 6;6(6):100994. doi: 10.1016/j.asmr.2024.100994 (PMC11701982; doi:10.1016/j.asmr.2024.100994)
Supplement: Appendix 1 [file mmc1.docx]

**Appendix 1**

**ICD-10 Laterality specific diagnostic codes key**

$ = “1” denoting right or “2” denoting left (i.e., M22.01– Recurrent dislocation of left patella)

# = “4” denoting right or “5” denoting left (i.e., S83.015A – Lateral dislocation of left patella, initial encounter)

? = “3” denoting right or “4” denoting left (i.e., T84.53XA - Infection and inflammatory reaction due to internal right knee prosthesis, initial encounter)

^ = “5” denoting right or “6” denoting left (i.e., L02.415 - Cutaneous abscess of right lower limb

& = “A” denoting Initial Encounter or “D” denoting Subsequent Encounter or “S” denoting Sequela (i.e., S83.015A – Lateral dislocation of left patella, initial encounter)

@ = “0” denoting right or “1”denoting left (i.e., T84.620 - Infection and inflammatory reaction due to internal fixation device of right femur)

* = “2” denoting right or “3” denoting left (i.e., T84.623 - Infection and inflammatory reaction due to internal fixation device of left tibia)

! = “A” denoting initial encounter for closed fracture or “B” denoting initial encounter for open fracture type I or II or “C” denoting initial encounter for open fracture type IIIA, IIIB, or IIIC or “D” denoting subsequent encounter for closed fracture with routine healing or “E” denoting subsequent encounter for open fracture type I or II with routine healing or “F” denoting subsequent encounter for open fracture type IIIA, IIIB, or IIIC with routine healing or “G” denoting subsequent encounter for closed fracture with delayed healing or “H” denoting subsequent encounter for open fracture type I or II with delayed healing or “J” denoting subsequent encounter for open fracture type IIIA, IIIB, or IIIC with delayed healing or “K” denoting subsequent encounter for closed fracture with nonunion or “M” denoting subsequent encounter for open fracture type I or II with nonunion or “N” denoting subsequent encounter for open fracture type IIIA, IIIB, or IIIC with nonunion or “P” denoting subsequent encounter for closed fracture with malunion or “Q” denoting subsequent encounter for open fracture type I or II with malunion or “R” denoting subsequent encounter for open fracture type IIIA, IIIB, or IIIC with malunion or “S” denoting sequela

% = “A” denoting initial encounter for closed fracture or “D” denoting subsequent encounter for fracture with routine healing or “G” denoting subsequent encounter for fracture with delayed healing or “K” denoting subsequent encounter for fracture with nonunion or “P” denoting subsequent encounter for fracture with malunion or “S” denoting sequela

**Patellar Instability:**

M22.0$ - Recurrent dislocation of patella, M22.9$ - Unspecified disorder of patella, M22.1$ – Recurrent subluxation of patella, M22.X$, - Patellofemoral disorder of knee , M22.3X$ - Other derangements of patella , M22.8X$ - Other disorders of patella, S83.00$& - Unspecified subluxation of left patella , S83.01$& - Lateral subluxation of patella, S83.01#& – Lateral dislocation patella, S83.09$& - Other subluxation of patella, S83.09#& - Other dislocation of patella, M25.36$ – Other instability of knee, M23.5$ – Chronic Instability of Knee, S8300#& – Unspecified Dislocation of Patella, M24.46$ - Recurrent Dislocation of Knee, M23.8X$ - Other internal derangements of knee, S83.8X$& - Sprain of other specified parts of knee, T84.02*& – Instability of internal knee prosthesis, S86.81$& - Strain of other muscle(s) and tendon(s) at lower leg level, S76.11$& - Strain of quadriceps muscle, fascia and tendon, M23.9$ - Unspecified internal derangement of knee, S83.10#& - Unspecified dislocation of knee

**Infection:**

M65.85$ - Other synovitis and tenosynovitis of thigh, M65.86$ - Other synovitis and tenosynovitis of lower leg, M01.X6$ - Direct infection of knee in infectious and parasitic diseases classified elsewhere, M00.06$ - Staphylococcal arthritis of knee, M00.86$ - Arthritis of knee due to other bacteria , M00.16$ - Pneumococcal arthritis of knee , T84.62@ - Infection and inflammatory reaction due to internal fixation device of femur, T84.62* - Infection and inflammatory reaction due to internal fixation device of tibia, M65.16$ - Other infective (teno)synovitis of knee , T81.31XA - Disruption of external operation (surgical) wound, not elsewhere classified, M0.09 – Pyogenic arthritis, M6505$ - Abscess of tendon sheath of thigh, M65.06$ - Abscess of tendon sheath of lower leg, M71.16$ - Other infective bursitis of knee , M71.56$ - Other bursitis, not elsewhere classified of knee, M25.56$ - Pain in knee , M25.86$ - Other specified joint disorders of knee, M79.81 - Nontraumatic hematoma of soft tissue, M25.46$ – Effusion of knee, M2516$ – Fistula of knee, S81.00$& - Unspecified open wound knee, S81.80$& - Unspecified open wound lower leg, T84.5?X& - Infection and inflammatory reaction due to internal knee prosthesis, L02.41^ - Cutaneous abscess of lower limb, M70.4$ - Prepatellar bursitis of knee, L03.11^ - Cellulitis of lower limb, M71.06$ - Abscess of bursa of knee, T8131X& - Disruption of external operation (surgical) wound, M96.840 - Postprocedural hematoma of a musculoskeletal structure following a musculoskeletal system procedure, S701$X& - Contusion of thigh, S800$X& – Contusion of knee, T8132X& - Disruption of internal operation (surgical) wound, T8142X& - Deep incisional surgical site infection following a procedure, M96.830 - Postprocedural hemorrhage of a musculoskeletal structure following a musculoskeletal system procedure, M72.6 - Necrotizing fasciitis, T84.59X& - Infection and inflammatory reaction due to other internal joint prosthesis, T81.30X& - Disruption of wound, M86.9 – Osteomyelitis, T847XX& -Infection and inflammatory reaction due to other internal orthopedic prosthetic devices, implants and grafts, M96.842 - Postprocedural seroma of a musculoskeletal structure following a musculoskeletal system procedure

**Stiffness**

M65.85$ - Other synovitis and tenosynovitis of thigh, M65.86$ - Other synovitis and tenosynovitis of lower leg, M24.56$ – Contracture of knee, M24.66$ – Ankylosis of knee, M25.76$ - Osteophyte of knee, M25.66$ - Stiffness of left knee, not elsewhere classified, M25.56$ - Pain of knee, M22.8X$ - Other disorders of patella

**Fracture**

S82.15$! - Displaced fracture of tibial tuberosity, S82.15#! - Nondisplaced fracture of tibial tuberosity, S82.14$! - Displaced bicondylar fracture of tibia, S82.14#! - Nondisplaced bicondylar fracture of tibia, S82.19$! - Other fracture of upper end of tibia, S82.10$! - Unspecified fracture of upper end of tibia, S82.16$% - Torus fracture of upper end of tibia, S89.01$% - Salter-Harris Type I physeal fracture of upper end of tibia, S82.00$! - Unspecified fracture of patella, S82.04$! - Displaced comminuted fracture of patella, S82.03$! - Displaced transverse fracture of patella, S82.09$! - Other fracture of patella, S82.03#! - Nondisplaced transverse fracture of patella, S82.02#! - Nondisplaced longitudinal fracture of patella, S82.04#! - Nondisplaced comminuted fracture of patella, S82.01#! - Displaced osteochondral fracture of patella, S82.02#! - Displaced longitudinal fracture of patella
